# A Brazilian Marseillevirus Is the Founding Member of a Lineage in Family *Marseilleviridae*

**DOI:** 10.3390/v8030076

**Published:** 2016-03-10

**Authors:** Fábio P. Dornas, Felipe L. Assis, Sarah Aherfi, Thalita Arantes, Jônatas S. Abrahão, Philippe Colson, Bernard La Scola

**Affiliations:** 1 Laboratório de Vírus, Departamento de Microbiologia, Instituto de Ciências Biológicas, Universidade Federal de Minas Gerais, Belo Horizonte, Minas Gerais 31270-901, Brazil; fabiopiod154@gmail.com (F.P.D.); felipelopesassis@gmail.com (F.L.A.); tsarantes@gmail.com (T.A.); jonatas.abrahao@gmail.com (J.S.A.); 2 Unité de Recherche sur les Maladies Infectieuses et Tropicales Emergentes (URMITE) UM63 CNRS 7278 IRD 198 INSERM U1095, Aix-Marseille Univ., 27 boulevard Jean Moulin, Faculté de Médecine, Marseille 13385, France; aherfi.s@gmail.com (S.A.); philippe.colson@univ-amu.fr (P.C.); 3 Fondation Institut Hospitalo-Universitaire (IHU) Méditerranée Infection, Assistance Publique-Hôpitaux de Marseille, Pôle des Maladies Infectieuses et Tropicales Clinique et Biologique, Centre Hospitalo-Universitaire Timone, Fédération de Bactériologie-Hygiène-Virologie, 264 rue Saint-Pierre, Marseille 13385, France

**Keywords:** Marseilleviridae, Marseillevirus, giant virus, Brazilian marseillevirus, lineage D, genomic analyses

## Abstract

In 2003, *Acanthamoeba polyphaga mimivirus* (APMV) was discovered as parasitizing *Acanthamoeba*. It was revealed to exhibit remarkable features, especially odd genomic characteristics, and founded viral family *Mimiviridae*. Subsequently, a second family of giant amoebal viruses was described, *Marseilleviridae*, whose prototype member is Marseillevirus, discovered in 2009. Currently, the genomes of seven different members of this family have been fully sequenced. Previous phylogenetic analysis suggested the existence of three *Marseilleviridae* lineages: A, B and C. Here, we describe a new member of this family, Brazilian Marseillevirus (BrMV), which was isolated from a Brazilian sample and whose genome was fully sequenced and analyzed. Surprisingly, data from phylogenetic analyses and comparative genomics, including mean amino acid identity between BrMV and other *Marseilleviridae* members and the analyses of the core genome and pan-genome of marseilleviruses, indicated that this virus can be assigned to a new *Marseilleviridae* lineage. Even if the BrMV genome is one of the smallest among *Marseilleviridae* members, it harbors the second largest gene content into this family. In addition, the BrMV genome encodes 29 ORFans. Here, we describe the isolation and genome analyses of the BrMV strain, and propose its classification as the prototype virus of a new lineage D within the family *Marseilleviridae*.

## 1. Introduction

Until recently, the concept of “giant viruses” was completely unrecognized. It emerged in 1982 after the discovery of *Paramecium bursaria* chlorella virus 1, which was classified in the *Phycodnaviridae* family that included giant viruses infecting algae [1]. In 2001, the *Phycodnaviridae* family was linked to other viral families including *Iridoviridae*, *Poxviridae* and *Asfarviridae*, which comprised a monophyletic group of viruses known as nucleocytoplasmic large DNA viruses (NCLDV) [2]. The concept of a giant virus dramatically expanded and gained notoriety in 2003 with the discovery of *Acanthamoeba polyphaga mimivirus* (APMV)*,* the prototype species of the *Mimivirus* genus, *Mimiviridae* family, isolated from the water of a cooling tower in Bradford, England by co-culturing with *Acanthamoeba polyphaga* [3]. Following the APMV discovery, dozens of members of this family were isolated, mostly from water, soil and, recently, from pneumonia patients, which confirmed previous evidence of their possible involvement in pneumonia [4,5,6,7,8,9,10]. Scientists were surprised by the mimivirus isolates due to their unique biological and molecular features, mainly their particle and genome sizes (up to 750 nm and 1.2 megabase pairs (Mbp), respectively). These were larger than those of small bacteria and their tremendous gene repertoires encoded proteins not previously identified in any virus, including some known as cellular hallmarks. In 2008 [4], La Scola *et al.* [4] reported the isolation of previously unknown icosahedral small viral particles, 50 nm in size, in virus factories and in the cytoplasm of cells infected by the APMV-like mamavirus strain. Given its functional analogy with bacteriophages, similar viruses were named virophages.

These observations revolutionized scientific knowledge and outlooks on viruses, including those on virus diversity and definition, and considerably fostered interest in the search for novel giant viruses. New giant viruses have been discovered in water and soil samples over the past decade using the same strategy of co-culturing with *Acanthamoeba* spp., which found five new recognized or putative viral families, including marseilleviruses [11], pandoraviruses [12,13], *Pithovirus sibericum* [14], faustoviruses [15] and *Mollivirus sibericum* [16]. Combined with data from metagenomics, these findings increasingly suggested that giant and large amoebal viruses were common and diverse inhabitants of our environment.

Marseillevirus was described in 2009 as a previously uncharacterized virus [11]. Marseillevirus particles are about 250 nm in diameter with icosahedral capsid morphology. Its genome comprises a circular double-stranded DNA molecule of 368,454 bp with a G+C content of 44.7%, encoding 457 putative proteins ranging from 50 to 1537 amino acids. Among these predicted proteins were 28 of the 41 core genes from the NCLDV. Phylogenetic studies revealed a distant relationship between Marseillevirus and other NCLDV families, suggesting the creation of a second family of giant amoebal viruses, for which Marseillevirus was the prototype member [11]. This new viral family was officially recognized by the International Committee on Taxonomy of Viruses (ICTV) in 2013 [17]. The monophyly of *Mimiviridae* and *Marseilleviridae* families and other NCLDV families, inferred from phylogenetic and phyletic analyses, as well as similarities in virion architecture and major biological characteristics for these viruses, led to a proposal to reclassify these viral families in a new viral order, the *Megavirales* [18].

Over the last six years, the *Marseilleviridae* family has expanded, and now nine members of this new family have been isolated from: (a) water from a cooling tower, river water, fountain water and human blood samples collected in France between 2005 and 2010; (b) water from a freshwater pond collected in Australia in 2014; (c) insect and fountain water samples collected in Tunisia in 2012; and (d) human stool samples collected in Senegal in 2012 [8,19,20,21,22,23,24,25,26,27]. Currently, the genomes of seven different members of the *Marseilleviridae* family have been fully sequenced and phylogenetic analysis of these viruses suggested the existence of three subgroups: Lineage A, consisting of Marseillevirus, Cannes8 virus, Senegalvirus and Melbournevirus; Lineage B, consisting solely of Lausannevirus; and Lineage C, consisting of Tunisvirus and Insectomime virus [28]. This was based on phylogenetic reconstructions carried out using core genes including the family B DNA polymerase, the VV A18 helicase, the D5 primase-helicase, the very late transcription factor 2B and the major capsid protein. Moreover, mean identity between orthologous proteins in members of a same lineage was ~97%, whereas lower mean identity (<73%) was observed among isolates from different lineages. No meaningful difference was observed regarding both amino acid and codon usage from Marseillevirus isolates, and the proposed pan-genome of the *Marseilleviridae* family was estimated to encompass 608 genes [28].

Marseilleviruses have been isolated from various samples collected in three countries from two continents. Because giant viruses from other families are widespread around the world, we believe that marseilleviruses can be found in as yet unexplored locations. Here, we describe the genome analyses of a Brazilian Marseillevirus (BrMV), the first Marseillevirus strain from the American continent, and propose that it is the prototype virus of a new lineage D within the *Marseilleviridae* family.

## 2. Materials and Methods

### 2.1. Virus Sample, Multiplication and Purification

BrMV was isolated in September 2014 from a sewage sample collected from a treatment station in the Pampulha lagoon in Belo Horizonte city, in the state of Minas Gerais in Brazil [29]. For multiplication of the virus, *Acanthamoeba castellanii* (strain NEFF) were multiplied in a 125 cm^2^ cell culture flask with 30 mL of peptone-yeast extract-glucose (PYG) medium at 28 °C. When the flasks contained a fresh monolayer of *A. castellanii*, they were infected with the isolated virus, and the flasks were kept at 30 °C for 72 h. After this, the cell lysates were collected and subject to purification [11]. For this, this material was filtered through a 0.8 μm and 0.45 μm filter to remove amoebal debris. The viruses were then ultracentrifuged at 22.000 rpm and the pellets were suspended in 1 mL of Page’s Amoeba Saline (PAS) solution. The suspension was again ultracentrifuged in a sucrose cushion (25%), and once more suspended in PAS solution. The purified virus was checked for the presence of bacterial contamination through inoculation in bacterial medium nonselective Luria broth (LB) agar plate and by Gram staining.

### 2.2. Analysis of Permissiveness of BrMV in Different Amoebae

To evaluate the replication profile of BrMV, the experiment was plotted on 96-well Costar^®^ microplates (Corning, NY, USA) containing 40,000 cells from different amoebae maintained in 100 µL of PAS culture medium per well. The amoebae used in this experiment were: *A. castellanii* (ATCC 30010), *A. castellanii* ALX (genotype T4, isolated from keratitis), *A. polyphaga* AR11 (genotype T4, environmental isolate from house dust), *A. polyphaga* (ATCC 30461, genotype T4), *A. polyphaga* (environmental isolate, genotype T4) and *A. polyphaga* (ATCC 30872, genotype T2). The cells were then infected with BrMv at an multiplicity of infection (MOI) of 0.01. After one hour of adsorption, the inoculum was removed and 100 µL of PAS was added per well. The microplates were maintained at 32 °C for 24 h and the cytopathic effects were then evaluated.

### 2.3. Genome Sequencing, Assembly and Annotation

The genome of BrMV was extracted using the automated EZ1 Virus Mini-Kit v.2 (Qiagen GmbH, Hilden, Germany) according to the manufacturer’s instructions. DNA quality and concentration were checked using a nanodrop spectrophotometer (Thermo Scientific, Waltham, MA, USA). Sequencing was performed using the Illumina MiSeq instrument (Illumina Inc., San Diego, CA, USA), with both paired end and mate pair applications, following the manufacturer’s protocol for library constructions. The sequence reads were assembled *de novo* using the ABYSS software [[30]). Gene predictions were performed using FgenesV [31], RAST (Rapid Annotation using Subsystem Technology) [32] and GeneMarkS [33] tools, and merged. Functional annotation was inferred by BLAST searches against the GenBank NCBI non-redundant protein sequence database (nr) (using an *e*-value <10^-5^ as threshold), the set of clusters of orthologous groups of proteins (COGs) of the NCLDV (named NCVOGs [34]) and by searching specialized databases using the Blast2GO platform [35]. Finally, the genome annotation was manually revised and curated. The predicted proteins that were smaller than 100 amino acids and had no hit in any database were ruled out. Those larger than 100 amino acids without hit in any database (so-called ORFans) were kept and analyzed using the PSI-BLAST tool to detect distant relationships with proteins available in the NCBI nr database [36].

### 2.4. Comparative Genomic and Pan-Genome Analysis

The genome synteny between the BrMV and other marseilleviruses was checked using the MAUVE program [37]. The Proteinortho tool [38] was used to define the strict core of *bona fide* orthologs shared among BrMV and amoebal marseilleviruses from lineages A–C, using the reciprocal best hit strategy with 10^−5^, 30% and 50% as thresholds for *e*-value, identity and coverage of amino acid sequences, respectively. In addition, we evaluated variations in the set of core genes (considering gene content from each new virus), and evaluated the intra and intergroup ratio of core genes/gene content. The OrthoMCL tool [39,40] was used to identify the paralogous gene families among all marseillevirus genomes which were analyzed. The average amino acid identity (AAI) calculator tool [41] was used to compare identity between orthologous genes from BrMV and other marseilleviruses, and from representative members of marseillevirus lineages. To estimate the size of the pan-genome of the *Marseilleviridae* family, their predicted proteins was clustered using the BLASTclust program [42] using an amino acid sequence identity of 30% and sequence coverage of 50% as thresholds. We also described pan-genome size variation by stepwise inclusion of each new virus annotation in the pairwise comparisons of the gene contents.

### 2.5. Phylogeny

We performed a hierarchical-clustering based on the gene presence/absence pattern of 5443 NCVOGs, using the MeV tool [43] with Pearson correlation as distance metric. The phylogenetic tree was visualized using the FigTree v1.4.1 tool [44]. In addition, the five *Megavirales* core genes, namely the family B DNA polymerase, the D6/D11 helicase, the VV A18 helicase, the D5 primase-helicase, and the Major Capsid Protein were used for the phylogenetic analyses. Amino acid sequences were aligned using the Muscle software [45]. Phylogenetic trees were built using the FastTree software [46] and the maximum likelihood method. The supertree was built using the five previously reconstructed phylogenetic trees (supplementary data), using the spr-supertree software [47]. The supertree algorithm was based on the subtree prune-and-regraft distance.

## 3. Results

### 3.1. Brazilian Marseillevirus

Isolation and identification of the BrMV have been already reported by Dornas *et al* [29]. In order to evaluate the cell permissiveness of BrMV, different amoeba cells were infected with BrMV for 24 h. Following infection, it was noted that no replication of BrMV took place in any *Acanthamoeba polyphaga* strain tested, and its replication was restricted to the *A. castellanii* tested lineages (Supplementary Figure S1).

### 3.2. Brazilian Marseillevirus Genome and Annotation

The BrMV genome (GenBank accession No.: KT752522) is a circular, double-stranded DNA molecule composed of 362,276 bp (Supplementary Figure S2). This is compatible with the genome sizes of the other marseilleviruses, which range from 346,754 bp (Lausannevirus; NC_015326.1) to 386,631 bp (Insectomime virus; KF527888). The mean G+C content of the BrMV genome is 43.3%, which is similar to that of other marseilleviruses. A total of 491 open reading frames (ORFs) were identified after merging all coding sequence predictions. These ORFs are fairly evenly distributed on both negative (261 ORFs) and positive (230 ORFs) strands, which was quite similar to gene distribution on the *Marseillevirus* genome (233 and 224 ORFs on negative and positive strands, respectively) [11].

The predicted ORFs ranged in size from 34 to 1553 amino acids, with an average length of 716 amino acids, which corroborates data from other genomes in the *Marseilleviridae* family [23]. The coding sequences exhibit a slightly higher mean G+C content of 44.3%, compared to 43.2% for non-coding sequences, with the same tendencies as other marseillevirus genomes (data not shown). This genome exhibits a gene density of 1.4 genes per kilo-bp, with a coding density of 97.1%, which is higher than the average (~90%) of family *Marseilleviridae* [28]. In addition, a total of 100 proteins are distributed into 30 paralogous families, of which the largest family consists of 14 MORN (Membrane Occupation and Recognition Nexus) repeat-containing proteins. Furthermore, we detected large paralogous families consisting of hypothetical proteins, in addition to families consisting of F-box-containing protein (six sequences), restriction endonuclease (five sequences), and putative Vsr/MutH/archaeal HJR family endonuclease (four sequences).

A total of 446/491 ORFs (90.8%) from the BrMV had significant BLASTp matches (coverage ≥ 50%; similarity ≥ 50%; *e*-value ≤ 10^-6^) to marseillevirus protein sequences available in the NCBI nr database, with a mean identity of 77.2%. The greatest number of best hits was with the Tunisvirus isolate, with 237 hits showing a mean identity of 73.2%. There were a lower number of best hit cases for the remaining marseilleviruses (mean: 52.5, range: 17 to 138), with lower mean identity values (mean: 58.8% ± standard deviation (SD): 8.7%; range: 55.9% to 71.0%). The number of best hits decreased with Insectomime virus, Lausannevirus, Cannes8 virus, Melbournevirus, Marseillevirus and finally Senegalvirus. Only 15 ORFs predicted in the BrMV genome showed identity values higher than 95% with marseilleviruses, namely Tunisvirus and Insectomime virus (group C). Moreover, only two ORFs (ORF364, a hypothetical protein; ORF381, a ubiquinone) predicted in the BrMV genome showed 100% identity with another marseillevirus, Lausannevirus (group B). A BLAST search against the NCBI nr database identified one sequence (ORF258—181 aa), with 50% coverage and 34% identity (*e*-value: 3 × 10^−8^ with a hypothetical protein of *Shrimp white spot syndrome virus*, which is not found in any other marseillevirus. Moreover, 16 putative ORFs (shorter than 100 amino acids (14 ORFs ranging from 50 to 100 aa)), in addition to 29 ORFs larger than 100 amino acids (ORFans), showing no homology with sequences available in GenBank databases, were identified. The absence of these ORFs in other marseilleviruses was confirmed by nucleotide search using the BLASTn tool. The BrMV gene content consists of 59.8% of hypothetical proteins (294 of the 491 predicted proteins). Table 1 describes the PSI-BLAST predictions for 23/29 ORFs tentatively classified as ORFans (79.3%) and highlights the presence of atypical viral proteins such as ORF 337, which was predicted to encode a cytochrome C-like protein, previously described in mimiviruses but not in marseilleviruses. Furthermore, we found that ORFs which were tentatively classified as ORFans could be predicted to encode a methyltransferase-like protein (ORF-L46), which may be associated with gene transcription regulation; a cysteine protease ATG4B-like protein (ORF-R86), which could be associated with cytoplasmic vacuole transport (Cvt) and/or autophagy; a cytidine and deoxy-cytidylate deaminase-like protein (ORF-L94), which is thought to be involved in the binding of the catalytic zinc ion; a protein-L-isoaspartate O-methyl transferase-like protein (ORF-R123), which may be involved in the repair and/or reduction of damaged proteins resulting from spontaneous decomposition of normal L-aspartyl and L-asparaginyl residues; a N-acetylneuraminic acid mutarotase-like protein (L324) which, in bacteria, accelerates the equilibration of the alpha- and beta-anomers of the sialic acid, N-acetylneuraminic acid, which in turn is used as a source of carbon; and others (Table 1). Neither aminoacyl-tRNA synthetases nor tRNA were found in the gene content of BrMV, as was the case for the other marseilleviruses.

Finally, BrMV was predicted to encode three histone-like proteins: (1) a histone H2A (L159) containing a C-terminal H2A-like histone fold and an unknown N-terminal domain; (2) a histone H2B/H2A fusion protein (L437) containing an N-terminal H2B-like and a C-terminal H2A-like histone; and (3) a histone H3 (R438) containing an N-terminal Histone-like transcription factor (CBF/NF-Y), an archaeal histone domain and a C-terminal H3-like domain. The histone-like proteins encoded by the BrMV resemble those predicted in other marseilleviruses, such as Lausannevirus [20].

### 3.3. Comparative Genome and Pan-Genome Analysis

**Figure 1:**
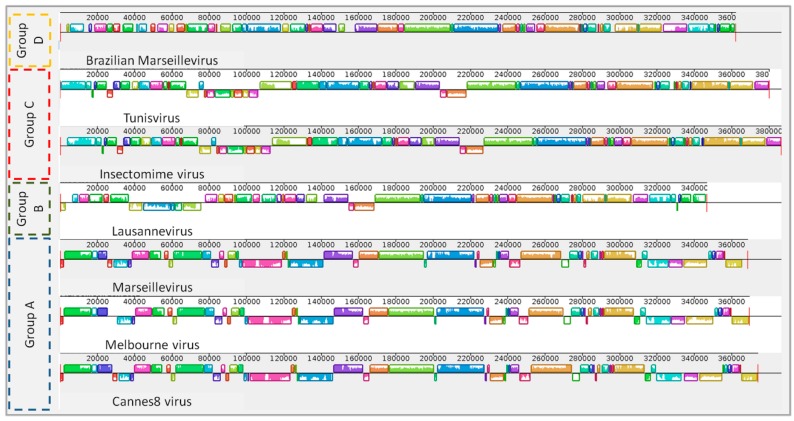
Genome alignment of BrMV and other marseillevirus strains. The figure shows genome architecture and synteny. Schematic genome alignment diagram was obtained using the MAUVE software package [37].

Genome synteny analysis of marseilleviruses showed that viruses from the same lineages display considerable conservative genome structure when compared to viruses from other lineages (Figure 1). Curiously, BrMV displayed a singular genome structure, with several rearrangements along its genome when compared to other marseilleviruses. Despite its low synteny with other marseilleviruses, the BrMV genome was more similar to Lausannevirus, from lineage B, and more dissimilar to viruses from lineage A, which presented several regions without homology with BrMV and other analyzed viruses (Figure 1).

**Figure 2:**
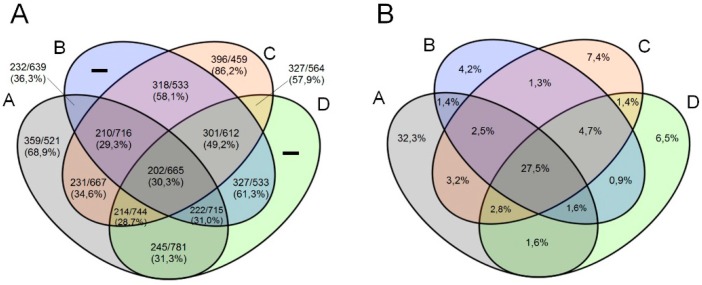
Distribution of orthologous gene clusters among marseillevirus lineages (**A**) and the proportion of orthologous gene clusters shared between and within lineages (**B**). For these analyses, we used the genome content of viruses from lineage A (Marseillevirus, Cannes8 virus, Melbournevirus and Senegalvirus), lineage B (Lausannevirus), Lineage C (Tunisvirus and Insectomime virus), and Lineage D (BrMV). The letters at each ellipsis’s top are related to the marseillevirus lineages A–D.

In addition, we observed a higher proportion of orthologous genes shared by marseilleviruses from same lineages than with marseilleviruses from different lineages (Figure 2A,B). The lineages B, C and D showed similar proportions of exclusive orthologous genes clusters. It is worth mentioning that exclusive clusters of lineages B and D are comprised by paralogous genes.

**Figure 3:**
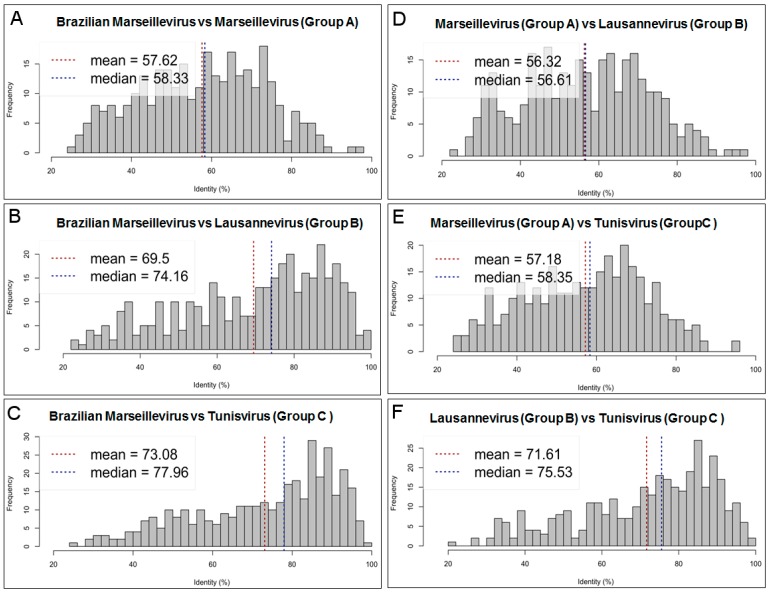
Average amino acid identity. In this analysis, estimates were reached using both best hits (one-way AAI) and reciprocal best hits (two-way AAI) between two datasets of proteins from the BrMV isolate and representative strains from marseillevirus groups (**A**–**C**). Plots (**A**–**C**) demonstrate the amino acid comparison between BrMV and marseilleviruses from groups (**A**–**C**); Plots (**D**–**F)** compare marseilleviruses from different lineages.

The BrMV amino acid sequences showed the lowest identity (Figure 3A–C) when compared to marseilleviruses from lineage A (mean: 57.6%; median: 58.3%), followed by those from lineage B (mean: 69.5%; median: 74.2%) and lineage C (mean: 73.1; median: 78.0%). Mean amino acid identity (AAI) was estimated for 311, 365 and 364 *bona fide* orthologous genes (reciprocal best hits) between BrMV and Marseillevirus, BrMV and Lausannevirus, and BrMV and Tunisvirus, respectively. Interestingly, we observed similar mean AAI values when comparing marseilleviruses from different lineages (Figure 3D–F), while the mean AAI values between viruses from the same lineages were 97% or higher. The mean AAI for orthologous genes (all best hits) shared by BrMV and other marseilleviruses was 11.7% lower than for *bona fide* orthologous ones, which is quite similar to values (11.1%) observed between marseilleviruses from different lineages.

Amino acid usages were also studied with a view to compare genes between BrMV and other marseillevirus strains, such as Marseillevirus, Melbournevirus, Lausannevirus, Tunisvirus and Insectomime virus, as well as with their amoebal host *A. castellanii*. Amino acid usage was very similar among the different marseilleviruses. However, some differences were observed between members from different lineages. BrMV showed singular usage for some codons and amino acids (e.g., codon TTC for amino acid phenylalanine; TTG and CTT for amino acid leucine). (SI Figure 3).

**Figure 4:**
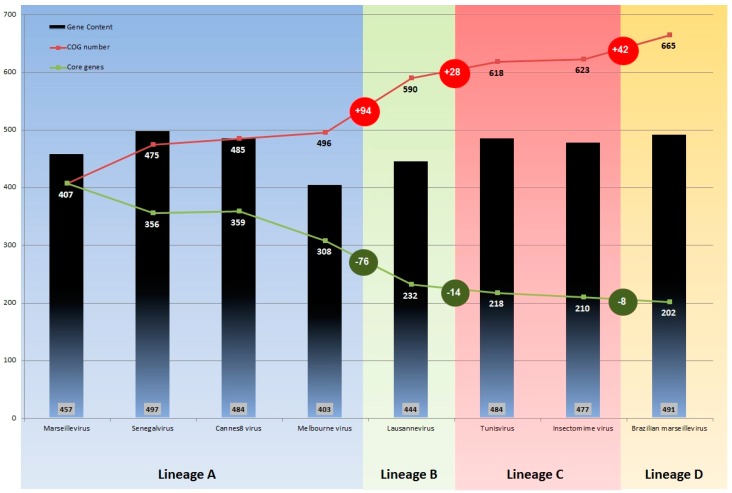
Pan-genome (**red line**) and core genome (**green line**) size of the *Marseilleviridae* family. Numbers into grey boxes refer to the gene number encoded by each virus strain. Numbers at line nodes represent the cumulative COG numbers after the inclusion of a new genome. Number in (**red and green**) circles demonstrate the variation of COGs after the inclusion of sequences from a different lineage. Colors on the graph identify viruses from the same lineage, alongside the proposed new lineage D consisting of BrMV.

Pan-genome analysis of all available sequences of marseilleviruses, carried out using the BLASTclust program, showed that pan-genome size increased with the addition of the BrMV gene repertoire. A total of 3737 proteins were grouped into 665 COGs (Figure 4), including 460 clusters consisting of at least two proteins from different marseillevirus strains and two clusters consisting of two paralogous proteins.

The largest COG was comprised of 146 proteins without predicted function (hypothetical proteins). The size of the pan-genome showed a continuous increase with the addition of the gene content of each newly discovered marseillevirus (Figure 4). It was noted that breaks occurred in this rising curve for each marseillevirus representative of a new lineages B and C, as is the case for BrMV; increments of 94 COGs from lineage A to B, and 28 COGs from lineages A and B to lineage C were found. Similarly, when BrMV was introduced, we observed an increment of 42 COGs in the pan-genome of the *Marseilleviridae* family. However, when we evaluated the core genome size variation, we observed an inverse profile, with an important decrease in the number of genes shared by the marseilleviruses from lineage A and lineages A plus B (−76 genes), then a slight decrease from lineages B to C (−14) and C to D (−8). Thus, no relevant break was observed on the core gene curve when BrMV sequences were included in the analysis (Figure 4), which reveals that, even if it corresponds to a putative new lineage, this isolate shares a very similar core gene set with other marseilleviruses.

### 3.4. Phylogeny

**Figure 5:**
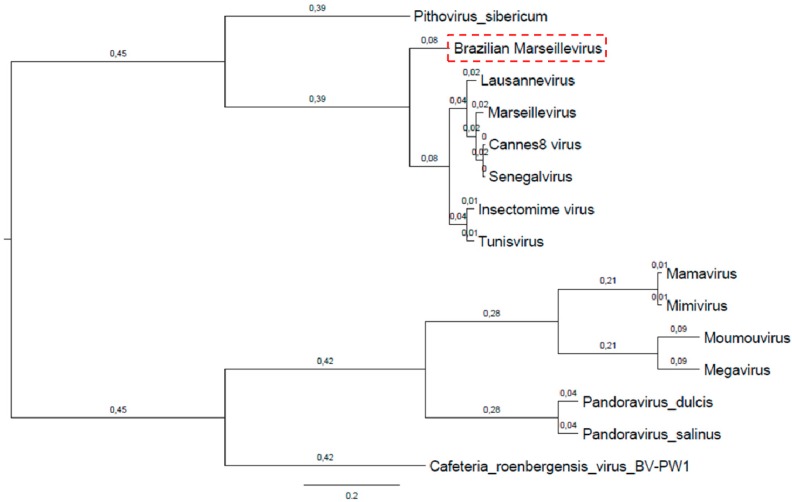
Hierarchical clustering tree based on phyletic patterns. Phylogeny based on the presence-absence matrix of 5443 NCVOG (clusters of orthologous genes shared by nucleocytoplasmic large DNA viruses). The Pearson correlation was used as metric distance, and the scale bar means the branch time.

A hierarchical clustering tree, based on the phyletic patterns, was constructed using a presence-absence matrix of 5443 NCVOG (clusters of orthologous genes shared by NCLDV). It shows that BrMV is apart from other known lineages. This analysis also shows lineage A to be closest to lineage B and distant to lineage C [37] (Figure 5).

**Figure 6:**
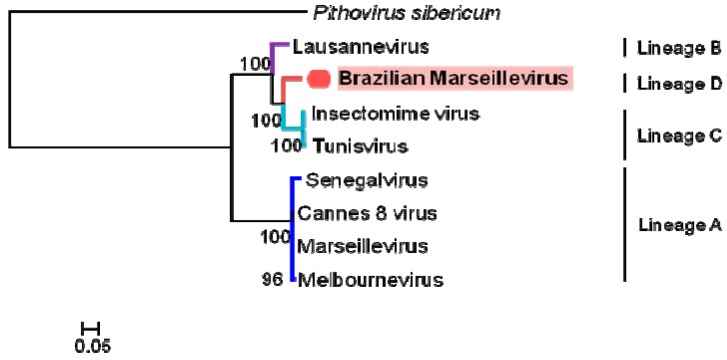
Phylogenetic reconstruction based on a concatenated alignment of the five core genes, DNA polymerase B, major capsid protein, VV-A18 helicase, D6/D11 helicase and D5 helicase. The amino acid sequences were aligned using Muscle and the tree was built using FastTree. *Pithovirus sibericum* was used as an outgroup. Branches delineating the different lineages of the family *Marseilleviridae* are colored (dark blue for lineage A, purple for lineage B, blue for lineage C and red for lineage D).

**Figure 7:**
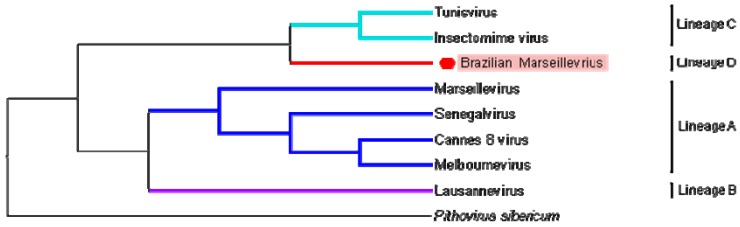
Supertree based on five phylogenetic trees. The five phylogenetic trees were built using FastTree and involved the DNA polymerase B, the major capsid protein, the VV-A18 helicase, the D6/D11 helicase and the D5 helicase amino acid sequences of the marseilleviruses and *Pithovirus sibericum*. The tree is rooted with *Pithovirus sibericum* used as an outgroup. Branches delineating the different lineages of the family *Marseilleviridae* are colored (dark blue for lineage A, purple for lineage B, blue for lineage C and red for lineage D).

Phylogenetic analyses based on core genes, DNA polymerase B, the VV A18 helicase, the D5 helicase, the D6/D11 helicase and the major capsid protein, for both concatenated alignment (Figure 6) and supertree (Figure 7) clearly delineate a first group consisting of what was previously known as lineage A, and consisting of Marseillevirus, Senegalvirus, Melbournevirus and Cannes 8 virus. Three other clades appear to delimit the phylogeny of the *Marseilleviridae* family, two composing the lineages previously known as B and C, and a third consisting of BrMV. Depending on the core gene studied, BrMV is clustered with Lausannevirus with low bootstrap values or delineates another clade.

## 4. Discussion

Isolation and identification of a new Brazilian Marseillevirus was performed using several techniques as previously reported in an environmental prospecting study [29]. Subsequently, genetic analyses were performed with a view to better characterize one more member of family *Marseilleviridae*. Surprisingly, this not only revealed a new Brazilian Marseillevirus, but also a new lineage of *Marseilleviridae*.

Through replication tests in *A. castellanii* and *A. polyphaga* using BrMV at MOI 0.01, we observed that the replication profile of BrMV is different from other marseilleviruses, such as Marseillevirus, a prototype of the *Marseilleviridae* family, and the Insectomime virus isolated from insect larvae (Supplementary Figure S1) [11,24]. These two members of *Marseilleviridae* family were isolated in *A. polyphaga* but can replicate in *A. castellanii*. In contrast, replication of BrMV is restricted to the tested *A. castellanii* strains and no cytopathic effects or viral titers were detected in the *A. polyphaga* cells infected with this virus. When comparing the replication profile of BrMV and the Marseillevirus prototype in *A. castellanii* cells, we observed similarities in terms of replication and the achieved viral titers (data not shown).

The BrMV genome has the second largest gene content of the *Marseilleviridae* family. Moreover, its genome was predicted to encode 29 new ORFans, defined as ORFs without detectable homology, although six of them were absent only from the predicted repertoires of gene products of other marseilleviruses. Moreover, we identified 16 ORFs less than 100 aa in length (pseudoORF), without detectable homology with any sequence in the NCBI nr database. The presence of such short pseudoORF may be a mere annotation artefact, or may represent novel short transcripts. We had identified 47 short ORFs encoding less than 100 amino acids with some correspondence in the NCBI nr database. It is worth mentioning that pseudoORF BrMV sequences were not identified in other marseillevirus genomes.

Furthermore, we used the PSI-BLAST tool to detect distant relationships between ORFans and proteins in the NCBI nr database. We were able to identify 23/29 (79.3%) putative homology for ORFans encoded by BrMV. These proteins, identified as encoded by marseilleviruses, have no described function yet, and were probably acquired by horizontal gene transfer HGT) events, which involved vertebrates, bacteria, fungi and viruses. This chimerical profile was previously described by Boyer *et al.*, (2009) [11], and probably results from the sympatric lifestyle of marseilleviruses, other giant viruses and microorganisms within amoebae.

In this paper, we propose the creation of a new lineage D in the *Marseilleviridae* family, of which BrMV would be the first member. This proposal is supported by comparative genomic analyses highlighting several divergences between BrMV and other marseilleviruses. First, genome synteny analysis showed a high level of structural conservation between viruses from the same lineages, while lower conservation was observed between viruses from different lineages. The BrMV genome displayed considerable differences, albeit they tended to be clustered in some regions, as previously described for other families of giant viruses such as poxviruses and mimiviruses [48,49]. Second, analysis of mean amino acid identity (AAI) showed identity values greater than 96% among orthologous genes of viruses from a same lineage, and identity values ranging from ~56% to ~71% among viruses from different lineages. The mean amino acid identity between BrMV and others marseilleviruses ranged from ~57% (lineage A) to ~73% (lineage C), suggesting that BrMV is distinct from the previously described lineages, and supporting its classification as a new marseillevirus lineage D. Third, codon and amino acid usage corroborates this hypothesis, given that some codons, such as TTC (phynelalanine), ATT (isoleucine), ACT (threonine) and others, could be used as lineage signatures, as a distinct usage of these codons was observed for BrMV. Fourth, looking at COG analyses among lineages B, C and BrMV, we noted a similar percentage of COGs shared between doublets B-C (58.1%), BrMV-B (61.3%) and BrMV-C (57.9%), and a higher proportion (86.2%) of COGs shared by lineage C viruses. This result highlights the similar distance based on COGs shared by different groups and BrMV, reinforcing the proposal to classify BrMV into a new marseillevirus lineage D. The proportion of all COGs generated by marseillevirus sequences corroborates this hypothesis, given that BrMV possesses similar amounts of unique COGs (6.5%) when compared with more closely related lineages B and C. Fifth, the pan-genome size of the *Marseilleviridae* family tended to increase with each new genome annotation. However, we observed a steep rise in the number of gene families from lineage A to B, followed by a moderate increase from lineage B to C with a tendency to remain stationary with the inclusion of sequences of other viruses from lineage C. In contrast, BrMV contributed to 42 new COGs that increased the pan-genome of marseilleviruses. This observation supports our hypothesis that BrMV is a member of a new lineage. It is worth mentioning that core genome analysis did not obviously distinguish BrMV from other lineages, highlighting that even when several exclusive genes were observed, BrMV shared a similar conserved gene content with others marseilleviruses.

Finally, phylogenetic analyses clearly delineated the *Marseilleviridae* lineage A, which is currently composed of the greatest number of viruses. Based on a concatenated alignment of five core genes, BrMV defines a fourth clade in the marseilleviruses phylogeny, beside lineage B consisting of Lausannevirus and lineage C consisting of the Tunisian marseilleviruses (Tunisvirus and Insectomime virus). However, it should be noted that, depending on the core gene studied, BrMV was clustered with Lausannevirus with a low bootstrap value, which is currently not sufficient to group them together in the same lineage. More remarkably, phylogeny based on gene presence/absence patterns of NCVOGs, which reflects the gene losses and gain history of the giant viruses, clustered BrMV into a distinct clade in the *Marseilleviridae* family.

Taken together, these data support the hypothesis of a fourth lineage consisting of BrMV. However, the current state of knowledge on marseilleviruses is certainly incomplete and, in the future, many other marseilleviruses may be discovered, leading to an ineluctable evolution in the current phylogeny of the marseilleviruses. Future data from new marseillevirus isolates may notably indicate whether these lineages can be classified as distinct viral species belonging to the *Marseilleviridae* family.

## 5. Conclusions

In summary, we isolated the first marseillevirus from the American continent. Genomic and phylogenetic studies indicate that this virus represents a new lineage, known as D, within the *Marseilleviridae* family. BrMV raises new questions about the diversity and ecological distribution of *Marseilleviridae*, highlighting the importance of prospective studies and pan-genomic analyses regarding this fascinating group of giant viruses.

## Figures and Tables

**Table 1:** Analysis of Brazilian Marseillevirus ORFans by Position-Specific Interative Basic Local Alignment Search Tool (PSI-BLAST)—ORFs with no significant similarity found are not presented.

ORFan ID	Protein Identification	Organism (1st and 2nd Best Hits)	Interation	Max Score	Total Score	Query Cover	*e*-Value	Ident	Accession Number
ORF_L46	Methyltransferase	*Rhizobium leguminosarum*	3	166	166	93%	4 × 10^−45^	12%	WP_025395836.1
	Methyltransferase	*Sinorhizobium meliloti*		165	165	93%	7 × 10^−45^	12%	WP_015242269.1
ORF_R48	Transglycosylase	*Streptomyces fulvoviolaceus*	2	256	256	93%	4 × 10^−78^	24%	WP_030603268.1
	Transglycosylase	*Streptomyces sp. WM6386*		229	229	93%	9 × 10^−68^	22%	WP_046261419.1
ORF_R84	hypothetical protein	*Aquimarina megaterium*	3	58,1	58,1	20%	8 × 10^−5^	18%	WP_025666489.1
	hypothetical protein	*Novosphingobium tardaugens*		83,5	83,5	20%	2 × 10^−15^	34%	WP_021691485.1
ORF_R86	cysteine protease ATG4B	*Apaloderma vittatum*	2	145	145	83%	10^−38^	23%	KFP75383.1
	cysteine protease ATG4B	*Tyto alba*		135	135	83%	10^−34^	23%	KFV59860.1
ORF_L94	cytidine and deoxycytidylate deaminase	*Acanthocystis turfacea Chlorella virus*	3	102	102	77%	2 × 10^−25^	24%	AGE49630.1
	cytidine and deoxycytidylate deaminase	*Acanthocystis turfacea Chlorella virus*		101	101	69%	7 × 10^-25^	23%	AGE55798.1
ORF_R115	DNA mismatch repair protein MutL	*Deinococcus deserti*	3	170	170	95%	2 × 10^−46^	18%	WP_012693648.1
	DNA mismatch repair protein MutL	*Deinococcus deserti*		145	145	80%	2 × 10^−39^	22%	WP_034401941.1
ORF_R123	protein-L-isoaspartate O-methyltransferase	*Frankia sp. CcI3*	2	124	124	64%	8 × 10^−31^	29%	WP_049761110.1
	protein-L-isoaspartate O-methyltransferase	*Frankia sp. BMG5.23*		124	124	64%	10^−30^	29%	WP_043591788.1
ORF_R124	ABC transporter substrate-binding protein	*Rheinheimera texasensis*	2	170	170	83%	10^−48^	29%	WP_031569037.1
	ABC transporter substrate-binding protein	*Vibrio gazogenes*		127	127	87%	2 × 10^-32^	23%	WP_027693958.1
ORF_L133	conserved signaling intermediate in Toll pathway	*Nannospalax galili*	2	142	142	87%	4 × 10^−37^	27%	XP_008831822.1
	conserved signaling intermediate in Toll pathway	*Nannospalax galili*		141	141	87%	7 × 10^−37^	27%	XP_008831824.1
ORF_R218	diguanylate phosphodiesterase	*Vibrionales bacterium SWAT-3*	3	147	147	70%	3 × 10^−38^	18%	WP_008217346.1
	diguanylate phosphodiesterase	*Vibrio crassostreae*		145	145	70%	8 × 10^−38^	19%	WP_048663292.1
ORF_R239	rho GTPase-activating protein 1	*Equus caballus*	2	107	107	41%	2 × 10^−24^	32%	XP_001490021.2
	rho GTPase-activating protein 1 isoform X2	*Equus caballus*		107	107	41%	2 × 10^−24^	32%	XP_005598135.1
ORF_L254	leucine-rich repeat-containing protein 9-like	*Lepisosteus oculatus*	2	130	130	64%	2 × 10^−31^	28%	XP_006632383.1
	Peroxidase	*Actinomyces sp. oral taxon 171*		122	122	64%	10^−30^	29%	WP_009394707.1
ORF_L292	coiled-coil and C2 domain-containing protein 1A isoform X5	*Papio anubis*	3	112	112	62%	10^−25^	29%	XP_009191945.1
	coiled-coil and C2 domain-containing protein 1A isoform X8	*Cercocebus atys*		112	112	62%	10^−25^	29%	XP_011949500.1
ORF_L300	ATP-dependent helicase	*Oenococcus oeni*	3	120	120	96%	3 × 10^−28^	20%	WP_002822412.1
	ATP-dependent helicase/nuclease subunit A	*Fructobacillus ficulneus*		117	117	94%	7 × 10^−28^	14%	GAO99721.1
ORF_R303	glycoside hydrolase family 9	*Ruminiclostridium thermocellum*	4	104	104	93%	4 × 10^−23^	26%	WP_023062725.1
	glycosyl hydrolase	*Ruminiclostridium thermocellum*		103	103	93%	6 × 10^−23^	24%	WP_020457778.1
ORF_R304	aggrecan core protein	*Callorhinchus milii*	3	118	696	100%	10^−27^	24%	XP_007906559.1
	aggrecan core protein	*Corvus brachyrhynchos*		103	926	100%	2 × 10^−22^	30%	XP_008638374.1
ORF_L309	peptide synthetase	*Microcoleus sp. PCC 7113*	6	424	424	97%	2 × 10^−136^	13%	WP_041780594.1
ORF_L324	N-acetylneuraminic acid mutarotase	*Vibrio variabilis*	3	212	212	78%	6 × 10^−63^	15%	WP_038216942.1
	N-acetylneuraminic acid mutarotase	*Vibrio sinaloensis*		211	211	78%	2 × 10^−62^	15%	WP_039481213.1
ORF_L337	cytochrome C	*Coleofasciculus chthonoplastes*	3	178	178	99%	2 × 10^−50^	17%	WP_006101072.1
	cytochrome C	*Mastigocladopsis repens*		177	177	99%	5 × 10^−50^	20%	WP_017318476.1
ORF_R351	extracellular dioxygenase	*Aspergillus kawachii*	3	151	151	61%	4 × 10^−39^	25%	CCX09620.1
	Intradiol ring-cleavage dioxygenase	*Penicillium expansum*		143	143	67%	3 × 10^−36^	17%	KGO45757.1
ORF_L367	regulator of telomere elongation helicase 1	*Cavia porcellus*	3	170	170	94%	10^−45^	17%	XP_013000054.1
	regulator of telomere elongation helicase 1	*Charadrius vociferus*		169	169	94%	2 × 10^−45^	18%	KGM00023.1
ORF_L375	putative protein binding surface, polypeptide binding	*Albugo laibachii Nc14*	4	137	137	90%	2 × 10^−36^	25%	CCA16909.1
ORF_R485	ephrin type-B receptor 4	*Nomascus leucogenys*	3	115	115	58%	7 × 10^−27^	21%	XP_012352012.1
	ephrin type-B receptor 4	*Microcebus murinus*		115	115	59%	9 × 10^−27^	21%	XP_012614574.1

## Supplementary Materials

The following are available online at www.mdpi.com/1999-4915/8/3/76/s1.
