# Supplementary material for: A Brazilian Marseillevirus Is the Founding Member of a Lineage in Family Marseilleviridae
Source: Viruses. 2016 Mar 10;8(3):76. doi: 10.3390/v8030076 (PMC4810266; doi:10.3390/v8030076)
Supplement: Supplementary file 1 [file viruses-08-00076-s001.pdf]

Supplementary Materials: A Brazilian Marseillevirus is the Founding Member of a Lineage in Family *Marseilleviridae*

Fábio P Dornas, Felipe L Assis, Sarah Aherfi, Thalita Arantes, Jônatas S Abrahão, Philippe Colson, Bernard La Scola

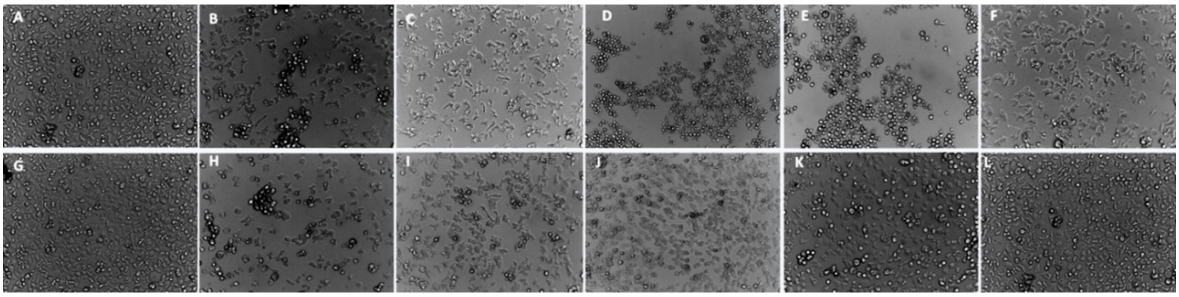

Figure S1. Analysis of permissivity to BrMV of different amoebae. Amoeba cells were infected with BrMV at MOI 0,01. The images represent the cytopathic effect of amoebae 24 h post-infection. (**A**) *A. polyphaga* (ATCC 30461, genotype T4); (**B**) *A. polyphaga* (environmental isolate, genotype T4); (**C**) *A. polyphaga* (ATCC 30872, genotype T2); (**D**) *A. castellanii* (ATCC 30010); (**E**) *A. castellanii* ALX (genotype T4, isolated from keratitis); (**F**) *A. polyphaga* AR11 (genotype T4, environmental isolate from house dust). Images **G**, **H**, **I**, **J**, **K** and **L** respectively represent the negative controls of amoebae used in the experiment. Magnification: 100×.

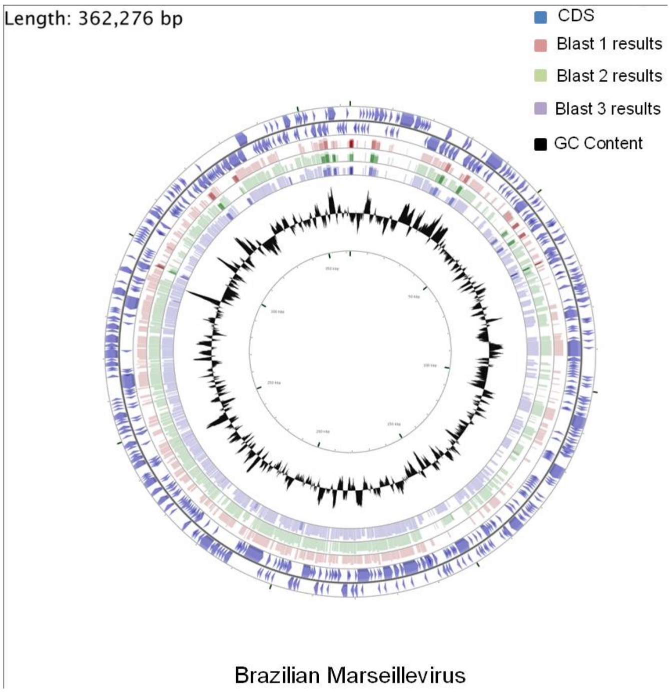

Figure S2. Genome map of Brazilian Marseillevirus. From the outside: circles 1 and 2 (clockwise and anticlockwise) show genes in positive strand and negative strand, respectively; circles 3 to 5 show the positions of BLAST hits detected by BLASTx (evalue = 10^{-6}; coverage and identity cutoff = 50%) searches against Marseillevirus (red), Lausannevirus (green) and Tunisvirus (blue); circle 6 depicts the G+C content; circle 7 depicts the genome size.

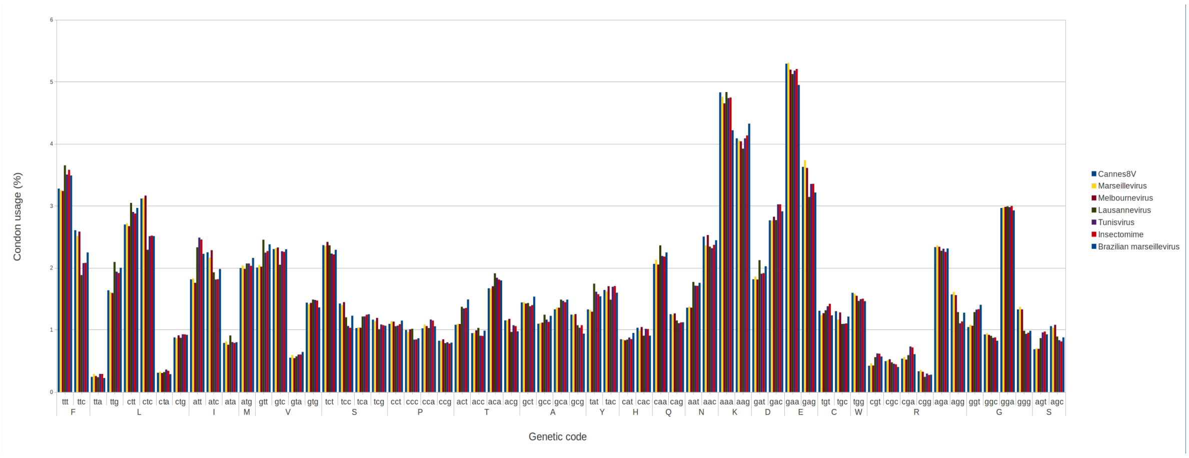

Figure S3. Amino acid and codon usages analysis. Amino acid and codon usages for BrMV genes and other Marseillevirus strains, such as Marseillevirus, Melbournevirus, Lausannevirus, Tunisvirus and Insectomime virus, as well as for their amoebal host *A. castellanii*.

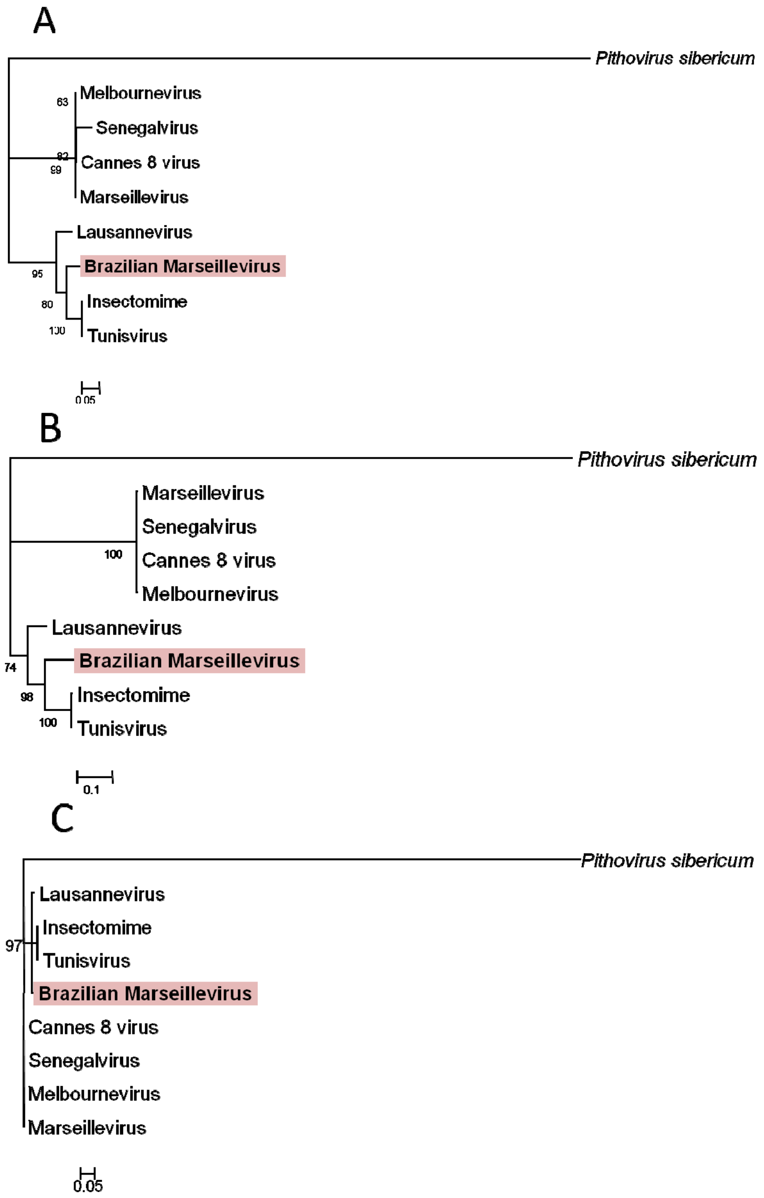

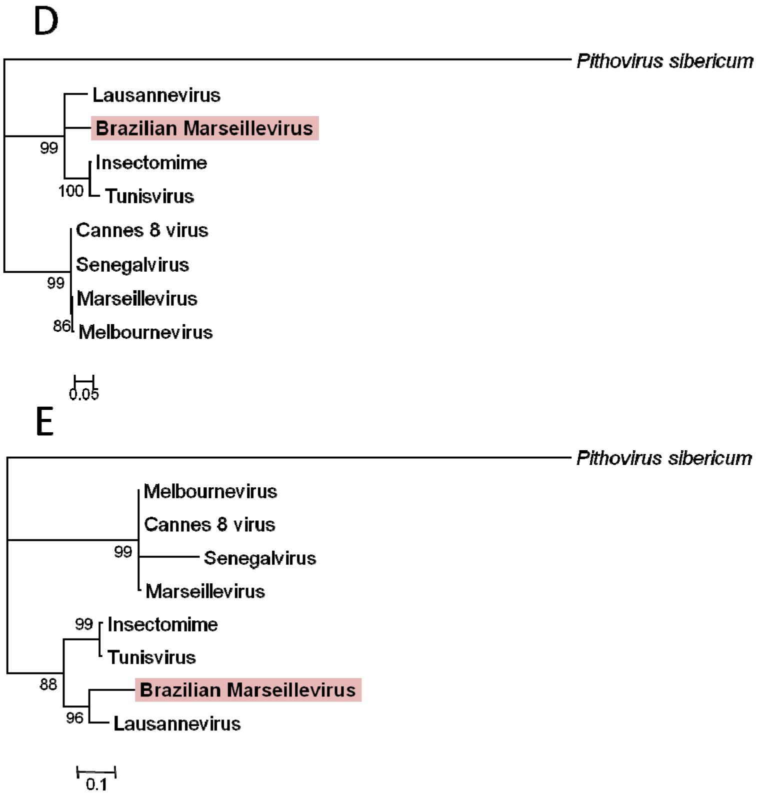

Figure S4. Maximum likelihood reconstruction based on amino acid sequences of conserved genes (**A**) D5 helicase; (**B**) D6-D11 helicase; (**C**) VV-A18 helicase; (**D**) DNA polymerase and (**E**) major capsid protein. The amino acid sequences were aligned using Muscle and the tree was built using FastTree. *Pithovirus sibericum* was used as an outgroup. The BrMV was highlighted in red.

© 2016 by the authors; licensee MDPI, Basel, Switzerland. This article is an open access article distributed under the terms and conditions of the Creative Commons by Attribution (CC-BY) license (http://creativecommons.org/licenses/by/4.0/).
